# Exploring the Relationship between Diamine Oxidase and Psychotropic Medications in Fibromyalgia Treatment, Finding No Reduction in Diamine Oxidase Levels and Activity except with Citalopram

**DOI:** 10.3390/jcm13030792

**Published:** 2024-01-30

**Authors:** Yaiza Tobajas, Marc Alemany-Fornés, Iris Samarra, Jordi Romero-Giménez, Jordi Cuñé-Castellana, Maria Tintoré, Antoni del Pino, Núria Canela, Josep M. del Bas, Nàdia Ortega-Olivé, Carlos de Lecea, Xavier Escoté

**Affiliations:** 1 Eurecat, Centre Tecnològic de Catalunya, Nutrition and Health, 43204 Reus, Spain; yaiza.tobajas@eurecat.org (Y.T.); jordi.romerog@eurecat.org (J.R.-G.); nadia.ortega@eurecat.org (N.O.-O.); 2 DR Healthcare-AB Biotek HNH, 43204 Reus, Spain; malemany@dr-healthcare.com (M.A.-F.); jordi.cune@abbiotekhealth.com (J.C.-C.); maria.tintore@abbiotekhealth.com (M.T.); carlos.delecea@abbiotekhealth.com (C.d.L.); 3 Centre for Omic Sciences (COS), Joint Unit URV-Eurecat, Unique Scientific and Technical Infrastructures (ICTS), Eurecat, Centre Tecnològic de Catalunya, 43204 Reus, Spain; iris.samarra@eurecat.org (I.S.); antoni.delpino@eurecat.org (A.d.P.); nuria.canela@eurecat.org (N.C.); 4 Eurecat, Centre Tecnològic de Catalunya, Biotechnology Area, 43204 Reus, Spain; josep.delbas@eurecat.org; 5 Department of Biochemistry and Biotechnology, Universitat Rovira i Virgili, Campus Sescelades, 43007 Tarragona, Spain

**Keywords:** fibromyalgia, DAO, histamine intolerance, psychotropic

## Abstract

**Background**: Histamine intolerance manifests when there is an imbalance between the production of histamine and the body’s capacity to metabolise it. Within the gastrointestinal tract, diamine oxidase (DAO) plays a pivotal role in breaking down ingested histamine. Insufficient levels of DAO have been linked to various diseases affecting the respiratory, cardiovascular, nervous, muscular, and digestive systems; some of these symptoms are evidenced in fibromyalgia syndrome. This underscores the crucial role of DAO in maintaining the histamine balance and highlights its association with diverse physiological systems and health conditions. The management of fibromyalgia commonly involves the use of psychotropic medications; however, their potential interactions with DAO remain not fully elucidated. **Methods**: This study delved into the influence of various psychotropic medications on DAO activity through *in vitro* experiments. Additionally, we explored their impact on the human intestinal cell line Caco-2, examining alterations in DAO expression at both the mRNA and protein levels along with DAO activity. **Results**: Notably, the examined drugs—sertraline, pregabalin, paroxetine, alprazolam, and lorazepam—did not exhibit inhibitory effects on DAO activity or lead to reductions in DAO levels. In contrast, citalopram demonstrated a decrease in DAO activity in *in vitro* assays without influencing DAO levels and activity in human enterocytes. **Conclusions**: These findings imply that a collaborative approach involving psychotropic medications and DAO enzyme supplementation for individuals with fibromyalgia and a DAO deficiency could offer potential benefits for healthcare professionals in their routine clinical practice.

## 1. Introduction

Fibromyalgia syndrome, also known as FMS, is a persistent ailment marked by musculoskeletal pain and associated symptoms stemming from factors released by immune cells [1,2]. This enduring condition encompasses widespread pain, insomnia, headaches, chronic musculoskeletal pain, muscle tension, diminished joint mobility, sleep disturbances, fatigue, mood disorders, cognitive dysfunction, anxiety, depression, and heightened sensitivity, impeding daily activities [3]. Affecting around 5% globally—predominantly women aged 30–35—fibromyalgia poses diagnostic challenges due to its intricate nature [4]. Research suggests that mast cells in the thalamus contribute to inflammation and pain by releasing histamine and several pro-inflammatory cytokines such as interleukin (IL)-1 beta (β), IL-6, and tumour necrosis factor alpha (TNF-α) [5]. Complementary treatments are sought by 90% of FMS patients, with non-pharmacological options preferred despite comparable efficacy [6]. Despite strides in understanding, accurate diagnosis and effective treatment remain elusive [7].

Histamine serves as a vital neurotransmitter, with diverse roles in biological functions encompassing inflammation, neural activity modulation, and immune response regulation [8]. The body naturally produces and acquires histamine from dietary sources. Elevated levels can cause histamine intolerance (HIT), resulting in various undesirable symptoms, including headaches [9,10]. Diamine oxidase (DAO) is an enzyme mainly located in the gastrointestinal tract that primarily metabolises dietary histamine, and DAO deficiency is a key HIT cause [11]. Insufficient DAO levels, as reported by the International Society of DAO Deficiency, impact multiple body systems, including respiratory (nasal congestion and asthma) [12], cardiovascular (hypotension, hypertension, and arrhythmias) [13], central nervous (hangover-like sensations and dizziness) [14], digestive (irritable bowel syndrome, constipation, stomach pain, and vomiting) [15], muscular (potential link to fibromyalgia and muscle pain) [3], and skeletal (potential osteopathic pain) [16] symptoms. HIT and DAO deficiency are associated with fibromyalgia, particularly in women with AOC1 (amine oxidase, copper containing 1, which codifies the DAO enzyme) gene variants, which are associated with a reduced DAO enzyme activity [3,17]. While a definitive cure does not exist, dietary adjustments and DAO enzyme supplements help to manage symptoms and improve the quality of life for those with HIT or DAO deficiency [18]. In addition, DAO interactions with pharmaceutical drugs are crucial considerations in managing histamine-related conditions like fibromyalgia [19]; certain pharmaceutical drugs, like proton pump inhibitors (PPIs) [20] and non-steroidal anti-inflammatory drugs (NSAIDs) [21], have been reported to potentially inhibit DAO activity, impacting individuals with HIT and necessitating awareness of medication interactions [20,22]. This inhibition may contribute to histamine-intolerance-related symptoms, potentially impacting individuals with fibromyalgia. In addition, the specific drug interactions between DAO and pharmaceutical drugs commonly used for fibromyalgia management are not extensively documented. 

HIT and fibromyalgia share common traits, reflecting interconnected physiological processes [23]. Both conditions involve the dysregulation of histamine levels, contributing to symptomatology. In fibromyalgia, mast cells release histamine, contributing to widespread musculoskeletal pain, while in HIT, elevated histamine levels result from impaired metabolism [17]. Notably, both conditions exhibit overlapping symptoms such as headaches, muscle pain, and fatigue, suggesting a potential link in their pathophysiology. Additionally, histamine’s involvement in neurosensitisation in fibromyalgia aligns with its role as a neurotransmitter in various bodily functions affected by HIT [24]. The impact on the central nervous system contributes to shared symptoms like cognitive dysfunction, anxiety, and mood disorders [5]. Recognising these commonalities enhances understanding and may guide comprehensive approaches to diagnosis and management, emphasising the importance of addressing histamine-related pathways in both conditions.

The prevalence of DAO deficiency in fibromyalgia is a subject of growing interest within the medical community as emerging recent research suggests a potential connection between these two conditions [3]. DAO, an enzyme crucial for histamine metabolism, plays a pivotal role in regulating histamine levels in the body [10]. In fibromyalgia, individuals often experience chronic musculoskeletal pain and recent studies have identified a link between this condition and histamine dysregulation compared with the general population [3,17,25], indicating a potential association between the two diseases. Notably, DAO deficiency, characterised by insufficient levels or activity of this enzyme [26], can contribute to elevated histamine levels [9], fostering an environment conducive to inflammation and pain. The intricate relationship between DAO deficiency and fibromyalgia extends beyond musculoskeletal manifestations, with implications for various body systems, including the central nervous, cardiovascular, and digestive systems [27]. Understanding the prevalence of DAO deficiency in fibromyalgia opens avenues for the exploration of targeted therapeutic interventions that address histamine dysregulation. While further research is needed to establish a definitive causal link, the identification of DAO deficiency as a potential factor in fibromyalgia underscores the importance of considering histamine metabolism in the comprehensive understanding and management of this complex chronic pain syndrome. DAO supplementation presents a potential avenue to treat fibromyalgia and challenge chronic pain syndrome by addressing histamine dysregulation. Supplementation with diamine oxidase aims to enhance histamine metabolism, potentially reducing the symptoms associated with fibromyalgia [25]. However, studies with a larger sample size would be needed to validate the long-term efficacy.

Preventive treatments for fibromyalgia focus on managing symptoms and improving overall wellbeing [28]. These may include regular exercise, stress-management techniques, adequate sleep hygiene, and a balanced diet [29,30]. However, a personalised, multidisciplinary approach is often essential for effective fibromyalgia prevention. Fibromyalgia management often involves medications related to mental health or neurological conditions (antidepressants or anticonvulsants are also prescribed to reduce pain and enhance mood) [31,32,33] and their classifications reflect their mechanisms of action and therapeutic purposes. Thus, sertraline and paroxetine are used to tackle mood disturbances [34]; they are selective serotonin reuptake inhibitors (SSRIs) commonly prescribed for depression and anxiety [35]. Alprazolam or lorazepam, both benzodiazepines, are used for the short-term management of anxiety relief [36]. The overuse of benzodiazepines poses risks of dependence, addiction, respiratory depression, and overdose [37]. Pregabalin is an anticonvulsant used to treat neuropathic pain, fibromyalgia, and certain types of seizures, modulating nerve signals [38]. Citalopram, also an SSRI used for depression and anxiety, may also help with mood symptoms [39]. Therefore, it is crucial for patients with fibromyalgia to closely collaborate with healthcare providers as treatment plans are tailored to address specific symptoms. Regular monitoring ensures optimal effectiveness and minimal side effects due to interactions with other drugs or as a result of overuse. For example, sertraline may cause nausea, insomnia, and sexual dysfunction [40], while alprazolam and lorazepam may lead to drowsiness, cognitive impairment, and dependence with overuse [41]. Pregabalin’s side effects include dizziness and drowsiness [42], and overuse can result in dependence [43]. Paroxetine may cause nausea, a dry mouth, and sexual dysfunction [44], with an increased risk of bleeding associated with overuse [45]. Citalopram can induce nausea and insomnia [46]. The overuse of all these medications can have serious consequences, exacerbating their side effects, including the risk of developing tolerance, dependence, and addiction [47]. Careful monitoring by healthcare professionals is essential to mitigate risks and ensure optimal treatment outcomes. In contrast, abrupt discontinuation, especially with benzodiazepines [48], may lead to withdrawal symptoms. In addition, fibromyalgia polypharmacy poses risks due to potential interactions and side effects from multiple medications, impacting effectiveness and safety [49]. This scenario makes it imperative to manage symptoms with various drugs, highlighting the need for the careful selection and monitoring of medication as well as regular communication with healthcare providers to mitigate the associated risks.

Consequently, a deeper understanding of the potential interactions between DAO and these medications is crucial because it could provide new insights into fibromyalgia management and may offer alternative strategies to improve fibromyalgia treatment, minimising side effects and reducing the risk of medication overuse. Thus, in this work, we evaluated the effects of DAO with the more common drugs used to treat fibromyalgia (sertraline, alprazolam, lorazepam, pregabalin, paroxetine, and citalopram) for the first time. We conducted experiments to examine how DAO interacted with this battery of drugs, including *in vitro* experiments to directly assess DAO activity inhibition by these treatments. Furthermore, we scrutinised this interaction in the human intestinal cell line Caco-2, assessing alterations in gene expression, protein levels, and enzyme activity.

## 2. Materials and Methods

The studies conducted by Tobajas and collaborators comprehensively detail the materials and methods employed in this research [50,51] and they are briefly explained below. Details of the assessment of the suppression of diamine oxidase activity by antifibromyalgia medications, evaluation of diamine oxidase activity inhibition by metabolised antifibromyalgia medications, liquid chromatography–tandem mass spectrometry (LC-MS/MS), *in vitro* cell culturing and experimental treatments, isolation of RNA and quantitative polymerase chain reaction (RT-qPCR) analysis, extraction of proteins, Western blot analysis, and DAO activity within Caco-2 enterocytes are available in the Supplementary Materials.

### 2.1. Cell Viability Measurement 

Viability was measured using a 3-(4,5-dimethylthiazol-2-yl)-2,5-diphenyltetrazolium bromide (MTT) assay. A colorimetric assay was used to assess the cell metabolic activity and viability [2]. MTT was converted by viable cells into a purple formazan product and the amount of formazan formed was proportional to the number of viable cells. Cells were incubated for 24 h in the presence of three increased concentrations of the different treatments: sertraline at doses of 4.08, 5.72, and 7.35 µM [52]; alprazolam at concentrations of 1, 5, and 15 µM [53]; lorazepam at doses of 1, 25, and 50 µM [54]; pregabalin at concentrations of 2.5, 12.5, and 25 mM [55]; paroxetine at doses of 10, 30, and 50 µM [56]; and with citalopram at doses of 1, 15, and 50 µM [57]. Following a 24 h incubation period, the treatments were withdrawn and MTT was introduced into each well. The plates were then further incubated for an additional 2 h at 37 °C. After obtaining the formazan precipitate, it was resuspended in isopropanol. Absorbance measurements were recorded at 570 nm and 650 nm (as a reference). Viability percentages for the different treatments were calculated relative to the vehicle control-treated cells. DMSO served as a negative control for viability. Then, after establishing the most effective dose for each drug, Caco-2 cells were placed in 12-well plates for the subsequent assays. The cells were treated with the drugs for 24 h to evaluate the mRNA expression, protein expression, and DAO activity [50,58]. Additionally, aminoguanidine was included in the experimental setup because of its recognised function as an inhibitor of DAO activity [59,60]. 

### 2.2. Statistical Analysis 

For each determination, an initial exploration was carried out to rule out discrepant points within the groups. The Grubbs statistical test was used for this purpose, using GraphPad Prism 10 software (GraphPad Software, Inc., La Jolla, CA, USA) [61]. All data were expressed as the mean ± standard error of the mean (SEM). To analyse differences in the different parameters, a one-way variability analysis (one-way ANOVA) was used followed by Dunnett’s post hoc test, which was used to analyse the differences between every treatment and the vehicle mean. A probability level of *p* < 0.05 was defined as being statistically significant.

## 3. Results

### 3.1. Citalopram May Slightly Reduce DAO Activity, Whereas Pregabalin Increases It

**Figure 1:**
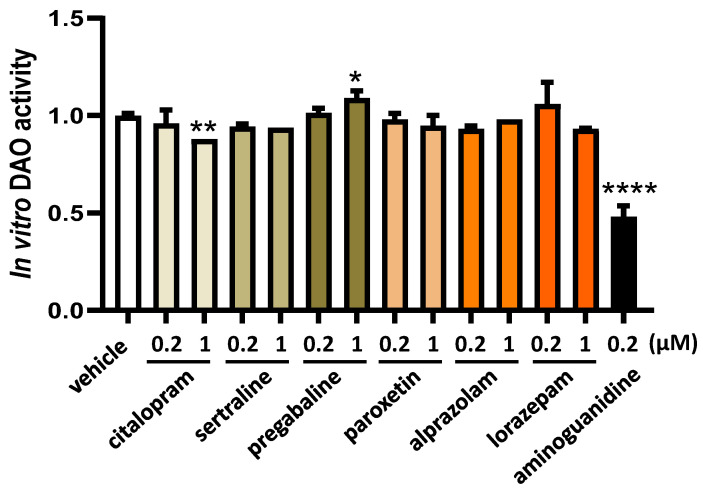
DAO activity was not affected by the presence of sertraline, paroxetine, alprazolam, and lorazepam at the indicated concentrations. In contrast, pregabalin induced DAO activity at higher assayed concentrations (1 µM), whereas citalopram reduced DAO activity at the highest dose tested (1 µM). Aminoguanidine produced an important reduction in DAO activity. Data are expressed as mean ± SEM. The results are expressed relative to the vehicle group. One-way ANOVA test followed by Dunnett’s post hoc test. * *p* < 0.05, ** *p* < 0.01, and **** *p* < 0.0001 vs. vehicle.

The *in vitro* LC-MS/MS chromatographic analyses revealed that the presence of sertraline, paroxetine, alprazolam, and lorazepam had no discernible impact on a reduction in DAO activity, as illustrated in Figure 1. This observation held true across both low and high concentrations for these antifibromyalgia treatments. Unexpectedly, when subjected to higher concentrations, pregabalin exhibited an augmentation in diamine oxidase (DAO) activity. Conversely, citalopram at higher tested concentrations and the positive control aminoguanidine led to a notable suppression in DAO activity (Figure 1).

**Figure 2:**
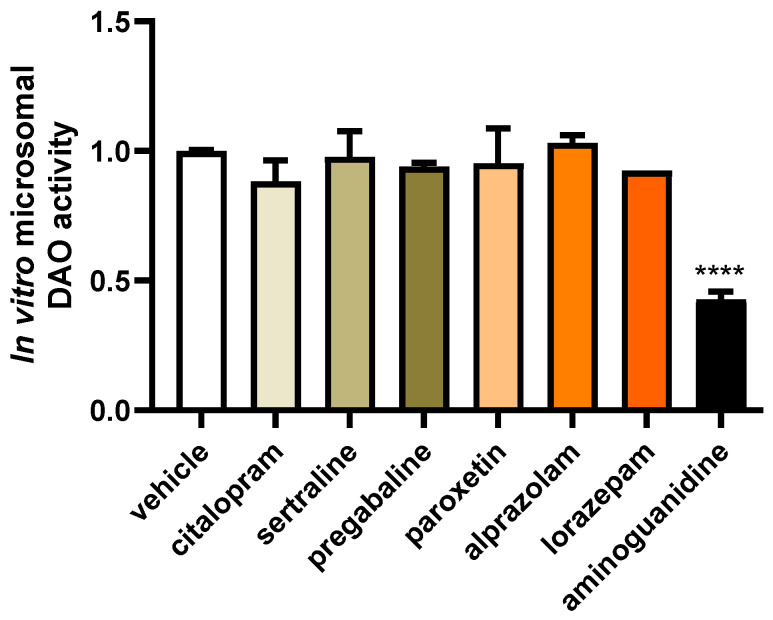
The incubation of microsomes with citalopram, sertraline, pregabalin, paroxetine, alprazolam, and lorazepam did not alter the activity of diamine oxidase (DAO). In contrast, aminoguanidine showed an important reduction in DAO activity. Data are expressed as mean ± SEM. The results are expressed relative to the vehicle group. One-way ANOVA test followed by Dunnett’s post hoc test. **** *p* < 0.0001 vs. vehicle.

To investigate the potential impact of changes to the chemical composition of common fibromyalgia treatment drugs during hepatic metabolism that could impact on DAO activity, we subjected these compounds to hepatic microsome incubation (Figure 2). Subsequently, DAO activity tests were performed to assess any alterations resulting from the exposure of these drugs to hepatic metabolism. This approach aimed to elucidate the potential influence of hepatic drug metabolism on the DAO function. No effects on DAO activity were observed after incubation with the metabolised citalopram, sertraline, pregabalin, paroxetine, alprazolam, and lorazepam drugs (Figure 2), whereas incubation with the positive control for inhibition, aminoguanidine, produced an important DAO activity repression. These findings indicated that the metabolic hepatic processes of the mentioned drugs did not adversely affect DAO activity, while aminoguanidine demonstrated its expected inhibitory effect.

### 3.2. Effects of Common Fibromyalgia Drugs on Intestinal DAO Activity

**Figure 3:**
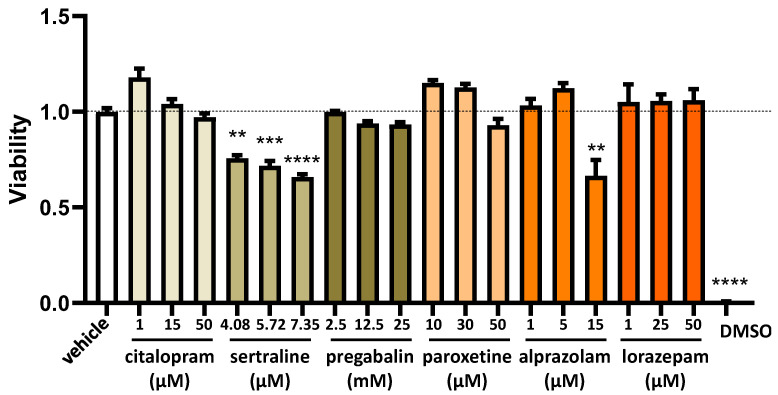
The viability of human enterocytes (Caco-2 cells) following treatment with specified concentrations of medications used for fibromyalgia: citalopram, sertraline, pregabalin, paroxetine, alprazolam, and lorazepam. Dimethyl sulfoxide (DMSO; 25%) served as a negative control to assess viability. The data, presented as mean ± SEM (*n* = 8), were normalised to the vehicle group. The statistical analysis included a one-way ANOVA test followed by Dunnett’s post hoc test. ** *p* < 0.01; *** *p* < 0.001 and **** *p* < 0.0001 vs. vehicle.

To evaluate the impact of varying concentrations of commonly used fibromyalgia treatments on the regulation of DAO in the human enterocyte Caco-2 cell line, we conducted cell viability MTT assays. These assessments covered a spectrum of drug concentrations encompassing low, moderate, and high doses. The aim was to comprehensively examine how different concentrations of fibromyalgia treatments might influence the viability of human enterocytes and, consequently, their potential effects on DAO regulation (Figure 3). No significant distinctions were observed in the impact on the viability of human enterocyte Caco-2 between common fibromyalgia drugs and the control vehicle (as depicted in Figure 3). This suggested a comparable influence on the viability of these cells, indicating that the tested fibromyalgia medications did not significantly alter cell viability compared with the control substance. The exception was sertraline, which was observed to have a cytotoxic effect with all of the tested doses in a dose-dependent manner, with a higher cytotoxicity at the highest dose (7.35 µM) (Figure 3). We selected the lowest concentration of sertraline (4.08 µM) based on the consideration that if modulation effects were observed on the levels of mRNA, protein, or DAO activity, these could be a consequence of the cytotoxicity induced by this compound. Similar cytotoxic results were observed with the higher dose of alprazolam analysed (15 µM) (Figure 3). Thus, the selected alprazolam concentration to perform the different analyses in this study was 5 µM. Regarding lorazepam, this compound did not present cytotoxic effects on Caco-2 at the tested doses. Therefore, the selected lorazepam concentration to perform the different analyses in this study was the higher tested, 50 µM. Similarly, pregabalin and paroxetine did not show cytotoxic effects on Caco-2 at the tested doses, but showed a clear tendency to decrease in viability at the 12.5 and 25 mM doses for pregabalin and 50 µM for paroxetine (Figure 3). Consequently, we selected a dose of 2.5 mM pregabalin and 30 µM paroxetine to carry out the subsequent experiments. Finally, citalopram did not present cytotoxicity at any of the analysed doses; however, the highest tested dose (50 µM) presented a tendency to be cytotoxic. Thus, a dose of 15 µM citalopram was selected to carry out the subsequent analyses.

**Figure 4:**
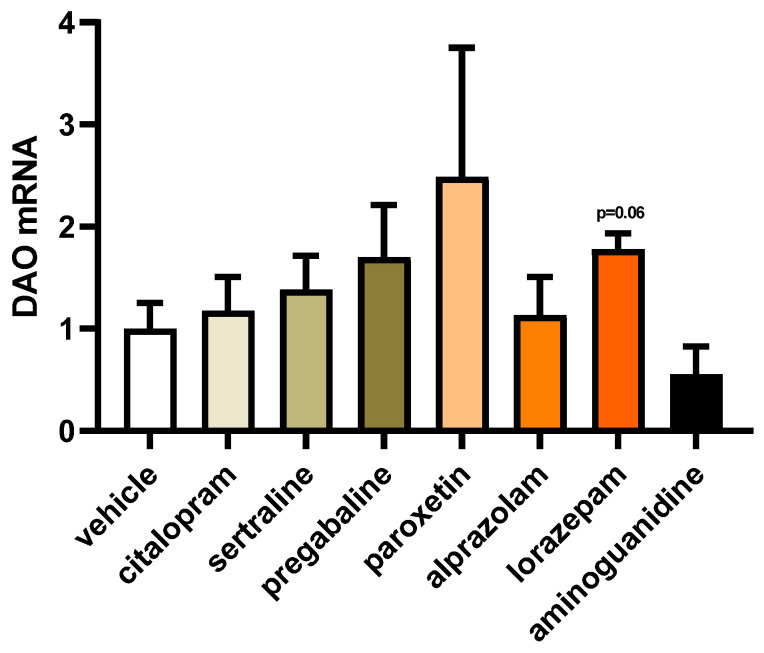
mRNA expression of DAO in human enterocytes (Caco-2) treated with antifibromyalgia treatments (citalopram, 15 µM; sertraline, 4.08 µM; pregabalin, 2.5 mM; paroxetine, 30 µM; alprazolam, 5 µM; lorazepam, 50 µM; and aminoguanidine, 90.5 mM). Data are expressed as mean ± SEM (*n* = 3). The results are expressed relative to the vehicle group. One-way ANOVA test followed by Dunnett’s post hoc test.

To explore the impact of frequently prescribed fibromyalgia treatments on the gene expression of DAO in human Caco-2 enterocytes, we conducted mRNA expression assays using a reverse transcription quantitative polymerase chain reaction (RT-qPCR). This approach aimed to elucidate how these medications might influence the transcriptional regulation of DAO in the Caco-2 cell line, providing insights into potential molecular mechanisms involved in DAO modulation (Figure 4). No discernible variations were detected in the mRNA expression of DAO in the enterocytes treated with the chosen antifibromyalgia drugs. Although a minor inclination towards increased DAO mRNA levels was noted following the treatment of Caco-2 enterocytes with lorazepam, the overall trend indicated that antifibromyalgia treatments did not significantly disrupt DAO mRNA expression in human enterocytes. These findings suggested a relatively stable influence of these medications on the transcriptional regulation of DAO in the tested cell line.

**Figure 5:**
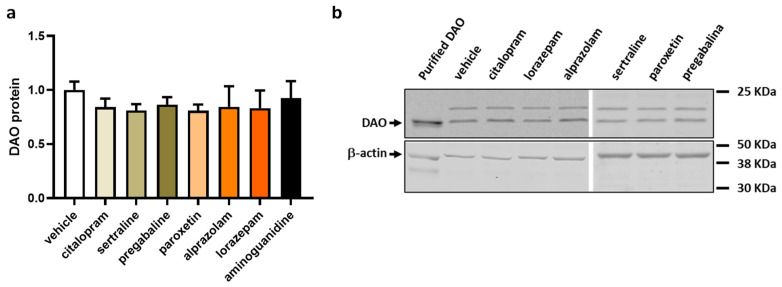
DAO protein levels in human colonocytes (Caco-2) treated with antifibromyalgia treatments (citalopram, 15 µM; sertraline, 4.08 µM; pregabalin, 2.5 mM; paroxetine, 30 µM; alprazolam, 5 µM; lorazepam, 50 µM; and aminoguanidine, 90.5 mM). (**a**) Densitometry analysis of relative DAO protein concentration after the indicated treatments. (**b**) Representative Western blot analysis of human DAO protein and β-actin levels shown as a housekeeping protein. The results are expressed relative to the vehicle group. Data are expressed as mean ± SEM (*n* = 3). One-way ANOVA test followed by Dunnett’s post hoc test.

To evaluate the influence of antifibromyalgia drugs on the protein expression of DAO in human Caco-2 enterocytes, we conducted protein expression assays utilising Western blot techniques. This investigative approach aimed to provide insights into how these drugs might affect the protein expression levels of DAO, contributing to a comprehensive understanding of their potential impact on the molecular regulation of DAO in the Caco-2 cell line (Figure 5). No alterations were observed in the protein expression of DAO in the enterocytes subjected to commonly prescribed fibromyalgia treatments, as illustrated in Figure 5. These results suggested that the medications intended to alleviate fibromyalgia symptoms may not significantly influence the regulation of intracellular DAO protein levels in human enterocytes. The stability of the DAO protein expression in response to these treatments implied that their primary therapeutic focus might not directly impact the intracellular regulation of DAO in the tested cellular context.

**Figure 6:**
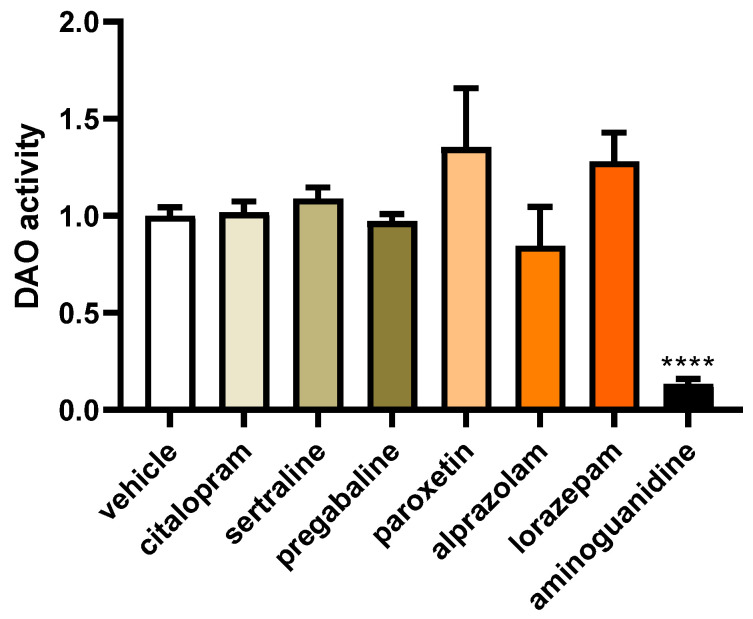
DAO activity in human enterocytes (Caco-2) treated with antifibromyalgia treatments (citalopram, 15 µM; sertraline, 4.08 µM; pregabalin, 2.5 mM; paroxetine, 30 µM; alprazolam, 5 µM; lorazepam, 50 µM; and aminoguanidine, 90.5 mM). Data are expressed as mean ± SEM (*n* = 3). The results are expressed relative to the vehicle group and relative to the amount of protein added in the assay. One-way ANOVA test followed by Dunnett’s post hoc test. **** *p* < 0.0001 vs. vehicle.

To investigate the impact of commonly prescribed antifibromyalgia drugs on DAO activity in human Caco-2 enterocytes, we employed a specialised DAO fluorescent activity assay. This method allowed us to discern any changes in the catalytic activity of DAO in response to the presence of these antifibromyalgia medications, providing valuable insights into their potential effects on histamine metabolism within the Caco-2 cell line (Figure 6). No notable differences were detected in the impact of the commonly prescribed antifibromyalgia treatments on DAO activity (Figure 6). However, consistent with the existing literature [59], a robust downregulation in DAO activity was evident in the enterocytes treated with aminoguanidine. These results suggested that medications with antifibromyalgia properties may not exert a significant influence on DAO activity in human enterocytes. 

## 4. Discussion

The current study has reinforced the notion that conventional therapies prescribed to alleviate fibromyalgia symptoms do not interfere with the functionality of DAO. These findings contribute to a growing body of evidence suggesting the compatibility of standard fibromyalgia treatments with the normal functioning of DAO. This was confirmed through an assessment of DAO activity in laboratory-based experiments as well as in a human intestinal epithelial cell line. It is noteworthy that lorazepam exhibited a distinctive characteristic by elevating the expression of DAO mRNA. Despite this increase, it did not correspondingly lead to an increase in DAO protein or activity. This indicated a subtle impact of lorazepam on the DAO system, underscoring the complexity of its effects on various regulatory aspects of DAO. In addition, citalopram appeared to physically interact with DAO at high doses, inhibiting DAO activity, as observed in the direct *in vitro* assay. Therefore, these results suggest that concomitant administration should be avoided and the doses for citalopram and DAO supplementation should be separated. However, the potential metabolisation of citalopram could reverse the inhibition observed in the *in vitro* assays, as seen in *in vitro* studies using microsomes and enterocytes. The findings of this study hold significance due to the escalating prevalence of fibromyalgia across diverse countries irrespective of gender, ethnicity, or economic status [62]. Consequently, there is a high likelihood that individuals with fibromyalgia may exhibit HIT or DAO deficiency. This underscores the relevance of exploring the relationship between fibromyalgia, HIT, and DAO deficiency, providing valuable insights into potential commonalities in these conditions among a broad spectrum of populations. In light of recent clinical data [25], incorporating DAO supplementation has emerged as a promising approach for individuals experiencing symptoms of fibromyalgia associated with DAO deficiency. This strategy aligns with existing pharmacological protocols, suggesting its compatibility and potential efficacy in addressing fibromyalgia symptoms among individuals with DAO insufficiency. The consideration of DAO supplementation adds a prospective dimension to the current therapeutic landscape for the management of fibromyalgia.

Nevertheless, it is crucial to emphasise that the nature of specific interactions can differ based on individual characteristics and the particular pharmaceutical drug involved. Therefore, the facultative must take into account these potential interactions when prescribing medications to individuals with HIT or DAO deficiency for fibromyalgia treatment [63]. A meticulous monitoring of their response to these treatments over time is essential. The positive impacts of DAO supplementation offer a promising avenue to mitigate the overuse of medications in fibromyalgia patients, thereby averting the risk of developing tolerance, dependence, and addiction [64]. 

In managing a chronic and incapacitating condition like fibromyalgia, adopting a gradual and phased escalation of medication is deemed preferable [65], mirroring the principles applied in pain therapy. This method entails initiating treatment with more physiological products and systematically integrating subsequent therapeutic interventions. The key aspect lies in the careful selection of treatments with non-interacting mechanisms and complementary actions, exemplified by the inclusion of DAO [25]. Furthermore, it is noteworthy that numerous psychotropic medications utilised in fibromyalgia lack officially approved indications and operate off-label [66], contributing to a complex web of potential interactions among these pharmaceutical agents. 

Nevertheless, integrating DAO supplementation as a complementary strategy for individuals grappling with fibromyalgia and HIT or DAO deficiency holds promise in mitigating symptoms linked to excessive medication use. The incorporation of DAO supplementation may contribute to a reduction in the requirement, dose, or duration of antifibromyalgia medications aimed at symptom management. Consequently, this has the potential to decrease the likelihood of encountering adverse side effects associated with prolonged or high-dose medication regimens [25]. This strategy of DAO supplementation harmoniously aligns with established pharmacological protocols, suggesting its compatibility and potential effectiveness in addressing fibromyalgia symptoms, specifically among those with DAO insufficiency. The consideration of DAO supplementation adds a prospective and complementary dimension to the existing therapeutic strategies for the management of fibromyalgia, offering a targeted and supportive avenue for individuals with identified DAO deficiency and reducing the risk of side effects in highly polymedicated chronic fibromyalgia patients. Supplementing with DAO in fibromyalgia patients has the potential to complement physiological processes and decrease reliance on other preventive measures or necessary doses to achieve the intended preventive effects.

This study presented several limitations that need to be considered. One primary constraint was that the behaviour of cultured cells in a laboratory setting may not precisely replicate the dynamics of cells within a living organism. A significant limitation of this study was that we could not evaluate all drugs used for different fibromyalgia symptoms, including amitriptyline and duloxetine. Another limitation of this study was the concentrations of sertraline tested, which, although based on previous *in vitro* studies [52], exhibited toxicity in this study; therefore, they should be tested at lower concentrations to assess their effects on DAO. Additionally, the cultured cells may have undergone gene expression or behavioural alterations due to the artificial conditions of their environment, including the absence of natural physical forces and the presence of growth factors different from those in living organisms. To address these limitations, future research should delve into preclinical animal models of DAO deficiency, closely mirroring the intricate microenvironment of living organisms. This approach will enable the evaluation of the long-term effects and chronic conditions of psychotropic treatments in conjunction with DAO supplementation. Moreover, the findings of this study should be validated through clinical trials involving fibromyalgia patients. 

## 5. Conclusions

The findings from the commonly prescribed antifibromyalgia-symptom drugs, including sertraline, pregabalin, paroxetine, alprazolam, and lorazepam, revealed no discernible inhibitory effects on DAO levels or activity, both in *in vitro* experiments and in human enterocytes cultures. However, it is noteworthy that citalopram exhibited a reduction in DAO activity in *in vitro* assays without translating to changes in DAO mRNA, protein levels, or activity in human cultured colonocytes. These findings suggest that a combined approach of prescribing antifibromyalgia medications alongside DAO enzyme supplementation for fibromyalgia patients with DAO deficiency may constitute a valuable strategy in fibromyalgia management. Additionally, it could contribute to preventing the risk of developing tolerance, dependence, and addiction to these drugs. Nevertheless, additional clinical studies are required to validate these findings.

## Data Availability

The data presented in this study are available on request from the corresponding author.

## Supplementary Materials

The supporting Materials and Methods information can be downloaded at: https://www.mdpi.com/article/10.3390/jcm13030792/s1, where all the materials and methods are explained in detail [67,68,69,70,71,72,73,74,75,76,77,78,79,80,81].
