# Supplementary material for: Exploring the Relationship between Diamine Oxidase and Psychotropic Medications in Fibromyalgia Treatment, Finding No Reduction in Diamine Oxidase Levels and Activity except with Citalopram
Source: J Clin Med. 2024 Jan 30;13(3):792. doi: 10.3390/jcm13030792 (PMC10856182; doi:10.3390/jcm13030792)
Supplement: Supplementary file 1 [file jcm-13-00792-s001.zip › jcm-2809887-supplementary.pdf]

Supplementary Materials and Methods

The materials and methods are essentially explained in Tobajas et *al*. [1,2] and they will be briefly presented below:

Assessment of the Suppression of Diamine Oxidase Activity by Psychotropic Medications.

Each of the psychotropic drugs were prepared and mixed with purified DAO obtained from porcine kidneys (0.1 mg/mL; Sigma-Aldrich, Germany) in phosphate buffer solution (PBS) at two final drug concentration (1 μM and 0.2 μM). Samples were placed in a water bath (1 h, 37 °C, 40 U/min) and 0.5 mL of 9 mM histamine were added to the sample. All the samples were kept in constant incubation, obtaining aliquots at different sampling times (t = 0, 0.5, 1, 1.5, 2, and 3 h) [3]. Perchloric acid was added to stop the enzymatic reaction. Internal standard (Histamine-d4) was also added. Samples were mixed and centrifuged at 15,000 rpm and the supernatant was diluted in 0.1 % formic acid in acetonitrile and transferred to vials for LC-MS/MS analysis. Two control samples were performed with DAO (0.1 mg/mL) and aminoguanidine (20 nM) in a DAO solution.

Evaluation of Diamine Oxidase Activity Inhibition by Metabolized Psychotropic Medications.

To understand whether modifications of citalopram, sertraline, pregabalin, paroxetine, alprazolam, and lorazepam produced effects on DAO inhibition, psychotropic drugs were treated with hepatic microsomes to simulate the metabolization of them by the liver [4]. For this, each of the psychotropicdrugs (citalopram 1 μM, sertraline 1 μM, pregabalin 1 μM, paroxetine 1 μM, alprazolam 1 μM, and lorazepam 1 μM) were incubated with microsomes (20 mg/mL; Fisher Scientific). The reaction was started with the addition of NADPH, incubating the samples for 1 h (37 °C, 40 U/min). Samples were centrifuged and the supernatant was used as the "test drug" for the enzymatic assay. The enzymatic reaction was started, incubated, sampling times, stopped and transferred to glass vials for LC-MS/MS analysis as explained in the *Analysis of DAO activity inhibition* section. Internal standard (Histamine-d4) was also incorporated [3].

Liquid Chromatography-Tandem Mass Spectrometry (LC-MS/MS)

The chromatographic separation was performed with a gradient detailed in the study by Tobajas *et al*. [1]. Mobile phase was water with ammonium formate and acetonitrile. The column temperature was set at 45 ºC. The source parameters applied operating in positive electrospray ionization (ESI+) are explained in the study by Tobajas *et al*. [1]. The MRM transitions used as well as the retention time for each compound are summarized in the methods section explained by Tobajas *et al*. [1]. The absolute value of the slope of the histamine consumption (30-120 min) represented in nmol was used to determine DAO activity in mU (nmol/min) and expressed relative to the vehicle group.

***In vitro* cell culturing and experimental treatments.**

The human colonic epithelial cell line Caco-2 (American Type Culture Collection, ATCC) was maintained at standard culture conditions in Dulbecco's Modified Eagle Medium (DMEM) containing glucose, inactivated foetal bovine serum (FBS), non-essential amino acids and penicillin–streptomycin. For all the experiments, cells were maintained for 14 days until enterocytes were completely differentiated [5–7]. Based on the concentration ranges for psychotropic drugs described in the literature [8–14] it was prepared a stock solution to treat the enterocytes considering the molecular weight, the dose of the active compound and the weight of a pill [1]. The pills were ground into a fine powder and powder drugs were solubilized with dimethyl sulfoxide (DMSO) as a carrier. Stock solutions were filtered and were stored until they use.

Isolation of RNA and Quantitative Polymerase Chain Reaction (RT-qPCR) analysis

Caco-2 homogenates were used for total RNA extractions using TriPure reagent (Roche Diagnostic, Barcelona, Spain) [30]. RNA concentration and purity were determined using a nanophotometer (Implen GmbH, München, Germany) and retrotranscribed to cDNA using the High-Capacity RNA-to-cDNA Kit (Applied Biosystems, Wilmington, DE, USA). The cDNAs were diluted before incubation with commercial LightCycler 480 Sybr green I master on a Lightcycler® 480 II (Roche Diagnostic). Primers were previously described in other studies and verified with Primer-Blast software (National Center for Biotechnology Information, Bethesda, MD, USA). Sequences of oligonucleotides used in this study were: DAO F1: 5'-CGCAGACGTGATTGTCAACT-3'; DAO R1 5'-GGATGATGTACGGGGAATTG-3' [15]; PGK1 F1: 5'-CAAGAAGTATCTGTCA-3'; PGK1 R1: 5'-CGAAGGTGGAAGAGTGGGAGTTG-3' [16].

Extraction of proteins and Western Blot analysis.

Fully differentiated enterocytes were treated for 24 h with the selected drugs and homogenized with lysis buffer (NaH_2PO_4, Na_2HPO_4, SDS, NaCl, NP40, NaF, sodium orthovanadate, PMSF, and protease inhibitor cocktail 1 (Millipore Sigma, Germany)) [1]. The protein extracts were quantified by the BCA method (Bio-Rad Protein Assay; BioRad, CA, USA). 25 μg of protein extracts were separated on SDS-PAGE and electroblotted to nitrocellulose membranes (LI-COR Biosciences, NE, USA) [30]. Efficient protein transfer was monitored by Ponceau-S stain. Next, membranes were blocked (5% BSA) and probed with primary antibodies against DAO (PA5-76708, Invitrogen, Carlsbad, CA, USA) and β-Actin (Santa Cruz Biotechnology, Inc.; TX, USA). Then, infrared fluorescent secondary antibodies anti-rabbit 680, anti-rabbit 800 and anti-mouse 680 (LI-COR Biosciences; 926-32211, 926-68071 and 926-68070, respectively) were used for detection and quantified using ImageJ [17].

DAO activity within Caco-2 enterocytes

DAO activity was measured with a Diamine Oxidase Activity Assay Kit (Sigma-Aldrich) [18]. Briefly, this assay provides a straightforward method to determine DAO activity of Caco-2 cell lysates. In the assay, DAO converts the provided substrate, yielding an intermediate and hydrogen peroxide (H_2O_2). H_2O_2 is then utilized by the DAO Enzyme Mix to generate fluorescence (excitation: 535 nm / emission: 587 nm) from the DAO Probe. Finally, the DAO activity was expressed relative to the amount of protein added in the assay. Thus, an aliquot of the enterocytes extracts was quantified by BCA.

[1] Tobajas Y, Alemany-Fornés M, Samarra I, Romero-Giménez J, Tintoré M, Del Pino A, et al. Interaction of Diamine Oxidase with Psychostimulant Drugs for ADHD Management. J Clin Med 2023;12. https://doi.org/10.3390/jcm12144666.

[2] Tobajas Y, Alemany-Fornés M, Samarra I, Romero-Giménez J, Tintoré M, Del Pino A, et al. Diamine Oxidase Interactions with Anti-Inflammatory and Anti-Migraine Medicines in the Treatment of Migraine. J Clin Med 2023;12. https://doi.org/10.3390/jcm12237502.

[3] Comas-Basté O, Latorre-Moratalla ML, Sánchez-Pérez S, Veciana-Nogués MT, Vidal-Carou MC. In vitro determination of diamine oxidase activity in food matrices by an enzymatic assay coupled to UHPLC-FL. Anal Bioanal Chem 2019;411:7595–602. https://doi.org/10.1007/s00216-019-02178-2.

[4] Knights KM, Stresser DM, Miners JO, Crespi CL. In Vitro Drug Metabolism Using Liver Microsomes. Curr Protoc Pharmacol 2016;74:7.8.1-7.8.24.

https://doi.org/10.1002/cpph.9.

[5] Yoshitomo A, Asano S, Hozuki S, Tamemoto Y, Shibata Y, Hashimoto N, et al. Significance of Basal Membrane Permeability of Epithelial Cells in Predicting Intestinal Drug Absorption. Drug Metab Dispos 2023;51:318–28. https://doi.org/10.1124/dmd.122.000907.

[6] Wu QY, Ma SZ, Zhang WW, Yao KB, Chen L, Zhao F, et al. Accumulating pathways of γ-aminobutyric acid during anaerobic and aerobic sequential incubations in fresh tea leaves. Food Chem 2018;240:1081–6. https://doi.org/10.1016/j.foodchem.2017.08.004.

[7] Guo XX, Zeng Z, Qian YZ, Qiu J, Wang K, Wang Y, et al. Wheat flour, enriched with γ-oryzanol, phytosterol, and ferulic acid, alleviates lipid and glucose metabolism in high-fat-fructose-fed rats. Nutrients 2019. https://doi.org/10.3390/nu11071697.

[8] Hao H, Wang G, Sun J, Ding Z, Wu X, Roberts M. Unidirectional inversion of ibuprofen in Caco-2 cells: developing a suitable model for presystemic chiral inversion study. Biol Pharm Bull 2005;28:682–7. https://doi.org/10.1248/bpb.28.682.

[9] Yu LS, Zhao NP, Yao TW, Zeng S. Zolmitriptan uptake by human intestinal epithelial Caco-2 cells. Pharmazie 2006;61:862–5.

[10] Stevenson CL, Augustijns PF, Hendren RW. Use of Caco-2 cells and LC/MS/MS to screen a peptide combinatorial library for permeable structures. Int J Pharm 1999;177:103–15. https://doi.org/10.1016/s0378-5173(98)00331-7.

[11] Durham PL, Russo AF. Regulation of calcitonin gene-related peptide secretion by a serotonergic antimigraine drug. J Neurosci Off J Soc Neurosci 1999;19:3423–9. https://doi.org/10.1523/JNEUROSCI.19-09-03423.1999.

[12] Siissalo S, Laine L, Tolonen A, Kaukonen AM, Finel M, Hirvonen J. Caco-2 cell monolayers as a tool to study simultaneous phase II metabolism and metabolite efflux of indomethacin, paracetamol and 1-naphthol. Int J Pharm 2010;383:24–9. https://doi.org/10.1016/j.ijpharm.2009.08.044.

[13] Ricchi P, Palma A Di, Matola T Di, Apicella A, Fortunato R, Zarrilli R, et al. Aspirin protects Caco-2 cells from apoptosis after serum deprivation through the activation of a phosphatidylinositol 3-kinase/AKT/p21Cip/WAF1pathway. Mol Pharmacol 2003;64:407–14. https://doi.org/10.1124/mol.64.2.407.

[14] Kulthong K, Duivenvoorde L, Sun H, Confederat S, Wu J, Spenkelink B, et al. Microfluidic chip for culturing intestinal epithelial cell layers: Characterization and comparison of drug transport between dynamic and static models. Toxicol Vitr an Int J Publ Assoc with BIBRA 2020;65:104815. https://doi.org/10.1016/j.tiv.2020.104815.

[15] Jagannath V, Marinova Z, Monoranu C-M, Walitza S, Grünblatt E. Expression of D-Amino Acid Oxidase (DAO/DAAO) and D-Amino Acid Oxidase Activator (DAOA/G72) during Development and Aging in the Human Post-mortem Brain. Front Neuroanat 2017;11:31. https://doi.org/10.3389/fnana.2017.00031.

[16] Panina Y, Germond A, Masui S, Watanabe TM. Validation of Common Housekeeping Genes as Reference for qPCR Gene Expression Analysis During iPS Reprogramming Process. Sci Rep 2018;8:8716. https://doi.org/10.1038/s41598-018-26707-8.

[17] Quesada-Vázquez S, Colom-Pellicer M, Navarro-Masip È, Aragonès G, Del Bas JM, Caimari A, et al. Supplementation with a Specific Combination of Metabolic Cofactors Ameliorates Non-Alcoholic Fatty Liver Disease, Hepatic Fibrosis, and Insulin Resistance

in Mice. Nutrients 2021;13. https://doi.org/10.3390/nu13103532.

[18] Beltrán-Ortiz C, Peralta T, Ramos V, Durán M, Behrens C, Maureira D, et al. Standardization of a colorimetric technique for determination of enzymatic activity of diamine oxidase (DAO) and its application in patients with clinical diagnosis of histamine intolerance. World Allergy Organ J 2020;13:100457. https://doi.org/10.1016/j.waojou.2020.100457.
